# Finding meaning: a realist-informed perspective on social risk screening and relationships as mechanisms of change

**DOI:** 10.3389/frhs.2023.1282292

**Published:** 2023-10-23

**Authors:** Arwen E. Bunce, Suzanne Morrissey, Jorge Kaufmann, Molly Krancari, Megan Bowen, Rachel Gold

**Affiliations:** ^1^ Research Department, OCHIN Inc., Portland, OR, United States; ^2^ Oregon Health & Science University, Portland, OR, United States; ^3^ Kaiser Center for Health Research, Portland, OR, United States

**Keywords:** realist evaluation, qualitative research, implementation research, social risk, social determinants of health, community health centers

## Abstract

**Background:**

Social risk screening rates in many US primary care settings remain low. This realist-informed evaluation explored the mechanisms through which a tailored coaching and technical training intervention impacted social risk screening uptake in 26 community clinics across the United States.

**Methods:**

Evaluation data sources included the documented content of interactions between the clinics and implementation support team and electronic health record (EHR) data. Following the realist approach, analysis was composed of iterative cycles of developing, testing and refining program theories about how the intervention did—or didn't—work, for whom, under what circumstances. Normalization Process Theory was applied to the realist program theories to enhance the explanatory power and transferability of the results.

**Results:**

Analysis identified three overarching realist program theories. First, clinic staff perceptions about the role of standardized social risk screening in person-centered care—considered “good” care and highly valued—strongly impacted receptivity to the intervention. Second, the physicality of the intervention materials facilitated collaboration and impacted clinic leaders' perception of the legitimacy of the social risk screening implementation work. Third, positive relationships between the implementation support team members, between the support team and clinic champions, and between clinic champions and staff motivated and inspired clinic staff to engage with the intervention and to tailor workflows to their settings' needs. Study clinics did not always exhibit the social risk screening patterns anticipated by the program theories due to discrepant definitions of success between clinic staff (improved ability to provide contextualized, person-centered care) and the trial (increased rates of EHR-documented social risk screening). Aligning the realist program theories with Normalization Process Theory constructs clarified that the intervention as implemented emphasized preparation over operationalization and appraisal, providing insight into why the intervention did not successfully embed sustained systematic social risk screening in participating clinics.

**Conclusion:**

The realist program theories highlighted the effectiveness and importance of intervention components and implementation strategies that support trusting relationships as mechanisms of change. This may be particularly important in social determinants of health work, which requires commitment and humility from health care providers and vulnerability on the part of patients.

## 1. Introduction

Evidence consistently shows that social context greatly impacts health (1–6). Structural stressors (7–10) often lead to the presence of social risks (such as housing and food insecurity and social isolation) that directly affect health (11–14). For the increasing number of health care organizations seeking to acknowledge and address these impacts, a necessary first step is awareness of a given patient's life circumstances. One approach to enabling such awareness is standardized social risk screening. To that end, numerous national health leaders in the United States (US)—including the Centers for Medicare and Medicaid Services (12), the National Association of Community Health Centers (15), the American Academy of Family Physicians (16), and the American Academy of Pediatrics (16, 17)—now recommend systematic social risk screening in primary care, often with an assumption of electronic health record (EHR) documentation of reported risks.

Despite increasing interest in social risk screening from payers, policymakers and care providers, it remains difficult for most clinics to embed systematic social risk screening into daily workflows. Although the 2019 Uniform Data System found that 71% of all Federally Qualified Health Centers (FQHCs) in the US reported collecting information on patients' social risks (18), in practice the collection of such information is rarely standardized or systematic. An analysis of 106 community-based health centers, the majority FQHCs, found that although 67% of the health centers had at least one social risk screen documented in the EHR between June 2016 and May 2018, the median number of screens per center was only 51 (19). Another analysis from this network found that between 2019 and 2021, only 2.6% of in-person and telehealth encounters led to EHR-documented food insecurity screening (20).

The trial described below (R18DK114701), which was conducted in this same network of community-based health centers and began in 2018, was designed to build knowledge to address this discrepancy between social risk screening intent and practice. It tested an approach to supporting the uptake of systematic social risk screening and EHR documentation, and sought to identify strategies that support embedding and normalizing social risk screening in clinic workflows. The analysis presented here reports the results of a realist-informed evaluation of how, why, for whom and under what circumstances this support intervention influenced social risk screening implementation and sustainment; results may advance understanding of how best to support those actions.

## 2. Materials and methods

### 2.1. Intervention

A pragmatic stepped-wedge trial tested the impact of a tailored five-step approach to implementing social risk screening and documentation in community-based primary care clinics across the US (21, 22). Although 31 clinics were recruited and randomized to the study (and therefore used for the stepped wedge analysis reported elsewhere) (22), five withdrew prior to their assigned intervention period. The remaining 26 clinics participated in cohorts of three to five sites for staggered six-month intervention periods between September 2018 and June 2021. Of note, the COVID-19 pandemic began to impact clinic operations halfway through the study, in March 2020. All study clinics were members of OCHIN, Inc., a non-profit health center-controlled network serving predominantly Federally Qualified Health Centers, whose members share a single instance of the OCHIN Epic EHR. The Kaiser Permanente Northwest Institutional Review Board gave ethics approval for the study.

The implementation support intervention included EHR training and change management coaching, as well as guides and workbooks to support decision-making at each step of the process. The choice of support strategies was guided by the four components of the Building Blocks of Primary Care model—engaged leadership, data (EHR)-driven improvement, empanelment and team-based care—and was intended to be pragmatic, sustainable and scalable. An implementation support team (IST) provided coaching and technical assistance throughout each intervention period. The composition of the IST underwent some changes as the team worked out their approach, but stabilized by the start of the third intervention period. The core IST members—an EHR trainer and practice coach—were both present during meetings with clinics while additional IST members (e.g., an Epic programmer, the study's principal investigator and project director) participated as pertinent. Clinics were asked to identify clinician and/or operational champions to support study work as it unfolded. Champions committed to spending a minimum of two hours a month working with the IST and encouraging social risk screening at their clinics. They were expected to attend all scheduled meetings and were welcome to invite additional clinic staff. Champions did not receive additional compensation for this work, although impact fees were paid to participating clinics.

Virtual check-ins between clinic champions and the IST were scheduled approximately twice a month throughout the six-month implementation period. Champions were asked to complete and return relevant workbooks prior to these meetings. Between check-ins the IST often communicated with clinic champions, usually by email, to follow-up on outstanding questions or requests, offer additional tips or resources, and sometimes to conduct additional training on specific EHR tools as requested by the clinic. The IST also coordinated virtual peer-to-peer conversations during each cohort. At the end of each intervention period the IST compiled a document for each clinic, called the SDH Summary, which detailed their decisions and plans for social risk screening implementation. The SDH Summary was implemented beginning with the second group of clinics.

### 2.2. Data sources

Quantitative data on social risk screening rates were derived from OCHIN's Epic EHR data. Qualitative data were the documented content of interactions between study clinics and the IST as described above, to limit the burden of research participation on clinics. The qualitative researchers sat in on many, but not all, of the IST's meetings with study clinics. Interaction content included recordings, transcripts and observation notes from the check-ins and peer-to-peer meetings, emails between the IST and study clinics, and transcripts from debriefs of the IST conducted by the qualitative researchers at the end of each intervention period. Additional data sources were a card study survey (reported elsewhere) (23, 24) and a short post-intervention champion survey. This survey requested reactions to a researcher-constructed summary of the factors that influenced the implementation of systematic social risk screening at the clinic. It also asked about any changes that impacted the clinic's ability to integrate social risk screening into care in the six months post-intervention. We received survey responses from half of the participating clinics. AB helped design the study and was involved from the start; SM joined near the end of the intervention periods and provided a fresh perspective on the data. A third qualitative researcher (IG) left midway through the study.

### 2.3. Theoretical framing

#### 2.3.1. Realist evaluation

The theory-driven realist approach asks not just whether an intervention was successful, but how and why it does or does not work, for whom and under what circumstances (25, 26). It assumes a stratified ontology in which reality consists of observable and unobservable phenomena across three levels: the empirical (events observed/experienced), the actual (that which is generated by the real but may not be observable) and the real (causal structures and mechanisms with enduring properties) (27–30). The goal of a realist evaluation is to uncover the causal links, known as mechanisms, between an intervention (here, the five-step tailored implementation support intervention) and an outcome (e.g., uptake of patient social risk screening) within a discrete context. Mechanisms—“the—often invisible—forces, powers, processes or interactions that lead to (or inhibit) change” (31)—are activated or repressed by pre-existing and unfolding structural, sociocultural and political economic conditions of intervention settings.

Mechanisms can be disaggregated into resource and reasoning (32). Mechanism: resource (M: resource) are program strategies intended to impact participant actions, which are always deployed within specific contexts (C). Mechanism: reasoning (M: reasoning) is the reaction and interpretation of participants in response to the interaction of M: resource plus context, which creates a program outcome (O). The relationship between these variables is conceptualized as *Mechanism: resources+Context –> Mechanism: reasoning=Outcome*, also known as CMO configurations (Box 1). The CMO heuristic is used to generate increasingly refined explanatory theories with interpretive validity—in this case, explanations of how and why the intervention impacted the uptake of social risk screening, for whom and in what circumstances. Here, realist evaluation helped us look for meaning in the beliefs and actions of participants in response to the introduction of the structured intervention. Analysis is composed of iterative cycles of developing, testing and refining these theories through engagement with study data as well as relevant insights from theory and published literature through a process of retroduction (33) to understand the complexities of implementation and identify subsequent actionable steps toward intervention goals (25, 34).

BOX 1 **CMO configurations (aka propositions, hypotheses, educated guesses) - a tool to keep focus on the realist causal explanatory framework.**

**CONTEXT (C):** broad conditions into which an intervention is introduced
**MECHANISM (M):** resources & reasonings that interact with each other and context to achieve changes
**OUTCOME (O):** Intended & unintended changes resulting from the intervention

Realist evaluation is methods agnostic, although in practice often qualitative forward because of the emphasis on context and participant experience. Methods and data sources are selected that are expected to explain how, why, for whom and in what circumstances a particular intervention does/does not support the intended change. As such, realist evaluation integrates well with pragmatic mixed method trials such as that in the current study. It also encourages applying other relevant theories to clarify and refine the specific initial program theories and enhance their explanatory power.

#### 2.3.2. Normalization process theory

Normalization Process Theory (NPT) is a middle range theory (a testable theory applicable to empirical investigation) that frames understanding of an intervention's material practices—what people are doing and saying as they interact with the program—as they unfold, under particular sociostructural circumstances. Its aim is to identify if and why an intervention took hold (35, 36). NPT categorizes the work of embedding and sustaining intervention practices into implementation contexts, implementation mechanisms, and implementation outcomes—each with associated constructs (37). As realist evaluation emphasizes generative mechanisms, we turned to the NPT implementation mechanism constructs to frame this analysis (Box 2). NPT theorizes that all implementation constructs must be effectively addressed and enacted to attain “normalization” or routinization of a practice, and in so doing provides a framework to understand why some practices are implemented and sustained while others are not (35).

BOX 2 **NPT implementation constructs.**

***Coherence*, or sense-making:** Do people individually and collectively agree about the purpose of the intervention, their role in it, and the value of it?
***Cognitive Participation*, or relational work:** Do people work together to create networks of participation and communities of practice around the intervention?
***Collective Action*, or enacting:** Do people collectively perform the implementation tasks required, and have adequate support?
***Reflexive Monitoring*, or appraisal:** Do people work together to appraise the intervention and its effects, and reconfigure intervention components or workflow in response?

(37, 38).

The constructs of Coherence and Cognitive Participation emerged as particularly pertinent to our understanding and the explanatory power of the realist program theories.

Coherence centers on how meaning and value—“sense-making”—are attached to an intervention by individuals working within a collective (i.e., does the work make sense to those involved?) (35, 39). It allows for attention to social actors as drivers of change, a central feature of realism, but expands beyond individual agency to include “extant vocabularies and repertoires of interaction, normative frameworks and belief systems, symbolic and material resources, power relations and legitimating authority—the key properties of collective action in social networks.” (35). Particularly relevant here are two coherence sub-constructs: internalization and communal specification. Internalization refers to the importance ascribed to an intervention by those who will potentially incorporate it into their work, while communal specification is about how those individuals come together to “collectively agree” that the intervention has purpose and value (37).

Cognitive participation reflects how relevant actors work together to create an environment within which a desired action will successfully take place (35, 37). The construct provides a set of questions about how people invest in processes of improvement. Its enrolment and legitimation sub-constructs were useful here. Enrolment speaks to how participants organize themselves to collectively contribute to the work. Legitimation refers to the creation of a community of practice by generating interest, motivation, engagement, and commitment, which then creates favorable conditions within which others in the group perceive the action to be of value (37).

### 2.4. Analysis

The primary quantitative outcome of the parent trial, previously reported (22), was the monthly clinic-level rate of social risk screening. Intervention effect was assessed using generalized linear mixed models (GLMM) which calculated the average difference between the pre-intervention period and both the 6-month intervention and post-intervention periods. These models compared clinics that had vs. had not yet received the intervention, and allowed estimation of the intervention effect to account for general time trends and clinic-level baseline covariates. In addition to this measure, clinic-specific raw 6-month average social risk screening rates were calculated for the realist-informed evaluation for the pre-intervention, intervention, and post-intervention periods.

As described above, we employed a realist-informed approach to investigate and analyze how and why the implementation support did/did not influence uptake and sustainment of social risk screening in varied contexts. At the end of each intervention period, the qualitative researchers created summaries for each clinic based on the realist framework. The clinic-specific summaries brought together qualitative data from all sources: transcripts, field notes and emails from IST-study team interactions, transcripts from debriefs of the IST, and the post-intervention survey These summaries were the start of the interpretive process, and a place to record clinic-specific initial ideas about how and why, for whom, and under what circumstances the implementation support drove social risk screening practices. Once all clinics completed their participation, AB and SM reviewed and discussed these summaries together, looking for similarities, patterns and outliers that, through an iterative process of immersion, reflection and discussion, led us to identify context-mechanism-outcome configurations and explanatory theories about the how and what of intervention effects. These theories were shared with the study project director (MK), an experienced qualitative researcher who had attended most clinic meetings, and whose perspective and suggestions refined the ideas. We then returned to the raw data to further query and hone our insights. The realist program theories were finalized before quantitative outcome data were available. Once the realist theories were established and the intervention's effect on screening rates was calculated, NPT was applied to the results to organize and enhance the explanatory power of the findings (40) as well as frame the program theories in a way that accounted for quantitative findings and supported transferability of lessons learned.

## 3. Results

Quantitative analysis indicated that although the rate of social risk screening was 2.45 times (95% CI 1.32–4.39) higher during the intervention period compared to pre-intervention rates, this impact was not sustained once the external implementation support ended (22). The realist program theories detailed below are organized by NPT construct to illuminate why the intervention as implemented was unsuccessful in normalizing systematic social risk screening in clinic workflows.

### 3.1. Coherence: care paradigms

When explaining their personal approach to care, individual providers and staff repeatedly noted that good care is first and foremost patient-centered and relational. This idea also underlies a collective vision of good care at the clinics. As one outreach manager and project champion explained, “I think it's really critical in this movement of social determinants of health and working with people, you get so busy and you have to slow down, you really do. That's why we’re in this work and do it is to really connect with people on a personal level vs. just kind of going through the motions.” This is the environment in which the implementation support intervention was introduced: individual and collective attitudes about the value of standardized social risk screening to patient-centered care had a direct impact on clinic engagement with the intervention. Clinics whose providers and staff saw social risk screening as a bridge to better patient relationships and care—i.e., who attached meaning to how standardization provided a structure that could support the work they were already doing and valued—were more likely to engage deeply with the intervention as a path toward systematic screening processes. Conversely, when providers and staff viewed the screening structure of yes/no answers about prescriptive domains of risk as an impediment to human connection it sometimes triggered a rejection of the entire process and intervention. The NPT construct of coherence helped explain participant reactions to structured screening in the context of existing care paradigms. It also provided insight into the degree to which the work was deemed meaningful by framing the ways in which individual feelings and approaches to care intertwine with clinic culture and priorities.

In realist terms, when standardized social risk screening (M: resource) was seen as contributing to better care (e.g., through improved communication and trust leading to more intimate conversations) and therefore aligned with care team members' expectations and understandings of good care (M: reasoning), then care teams invested time and effort into increasing the number of social risk screens (O). On the other hand, when standardization was seen as interfering with the patient-provider connection (M: reasoning), e.g., by necessitating recording pre-determined data rather than patient-prioritized concerns or by requiring more attention to a computer screen than to the patients, there was limited motivation to create or adopt systematic screening workflows (O).

Pragmatic constraints such as time, staff availability and alternative clinic priorities (C), staff feeling unprepared to engage in these potentially sensitive and difficult conversations (C), and settings with few clinic or community services to meet patient-reported needs (C) challenged intervention uptake even at clinics whose culture otherwise emphasized the value of social risk screening to patient care. Conversely, current or expected financial reimbursement for screening (C), existing workflows that easily accommodated social risk screening (C) and a clinic culture that valued quality improvement initiatives (C) created a supportive environment for staff to engage with the implementation support and adopt or increase rates of formal social risk screening.

The intervention encouraged coherence among study participants by fostering discussion about goals and motivations for social risk screening, and continually tying efforts to those motivations (41). As sense-making around social risk screening evolved during the intervention period, staff at some clinics experienced a cognitive shift from a mechanistic notion of screening for the collection of reportable data to screening as a way to improve clinical care. As one population health manager and project champion said, “my ulterior hidden motive was just to get some good data that we could use for our grant reports. But a lot of the providers have been really excited about it and since we've rolled it out they've seen the value in it. So that kind of is feeding into further success.” In addition to helping her be intentional in establishing feasible priorities and using data to inform and track SDH work, this champion also noted that where before there was resistance to screening for needs that the clinic lacked resources to solve, now care teams help however they can and refer if possible: “So we don't have to fix everything, but even just knowing about things sometimes is just that first step. Like just asking that question and being able to incorporate that into their care plan.”

### 3.2. Cognitive participation: workbooks

The physicality of the implementation support workbooks was an important (unanticipated) intervention component that facilitated cognitive participation. While originally intended as an organizing tool for clinic champion decision-making, the workbooks' materiality and step-by-step configuration created a structure around which staff from a variety of clinic roles and perspectives could discuss, dispute and ultimately coalesce around a shared vision and process. The workbooks thus provided a template for engaging and organizing their collective contributions (NPT cognitive participation sub-construct: enrolment).

In realist terms, the workbooks' materiality (M: resource) provided grounding for discussions of the social risk-related work and created a shared sense of purpose and direction (M: reasoning) among diverse actors. In turn, these actors engaged with the ideas and collaborative decision-making around goals and process (O1) that set the stage for implementation and potential sustainment of social risk screening (O2). Some of the clinics that used the workbooks as a collaborative tool had a pre-existing culture of cooperative decision-making and workflow development (C); for others the catalyst to use the workbooks to facilitate collective engagement was less clear. In contrast, clinics in which workbook completion was primarily the provenance of a single person fell mainly into two groups: (i) those whose champion had enough authority and investment in the idea of social risk screening to move the work forward on their own (C), and (ii) those in which social risk screening was not seen as enough of a priority to dedicate time from multiple staff (C).

Clinics that did use the workbooks to support a collaborative approach to goal setting and workflow development, approximately one quarter of the study sites, told us they found it valuable. One clinic champion, a social worker, noted: “I found it to be really helpful and interesting that we worked on it collaboratively to see each person on the team's perspective and talk out just really where we are at … Let's put it all on paper and look at it … it was helpful.” Another champion, a quality director, told us that she “really appreciated having those [workbooks] to guide those conversations and engage the group” and explained “we used this [workbooks] as a guide for discussion … Everybody looked at it in advance and then when we came together we filled it out as a group.”

The synchronous nature of such conversations appeared key to generating enthusiasm and cognitive participation. Some champions, unable to pull everyone together in real time, instead asked for separate feedback from multiple individuals. These champions noted that although it was useful to incorporate multiple perspectives into the plan, many felt the asynchronous process lacked the active engagement and back-and-forth interactions crucial to creating a shared sense of purpose and direction. As one champion, a social work supervisor, told us: “The missing part for me here again is … to be able to do this with the work group or group of people and have more conversation about it.” Another, a Community Health Worker (CHW) manager, said they had been working asynchronously but going forward wanted to “actively make sure that as a group we get together and go over the new tools, go over the workbook and really use everybody on our team to … really get a good idea as to what exactly we’re going to do.”

Clinics that did not use the workbooks to engage staff in designing goals and workflows sometimes spontaneously mentioned that doing so would have been beneficial, particularly at the beginning of the process. One champion, an EHR support analyst, observed: “I think the workbooks in the beginning probably would've been helpful had the whole team been assembled. So I guess the workbooks and what we're doing, why we're doing it, all of that. But make sure that whoever is going to be involved in it is there for that.” This emphasis on the need for collective participation in the “why we’re doing it” conversation(s) maps to comments made by many study participants, who consistently called out use of the workbook on identifying clinic goals as a key moment for collaboration. As one champion and CHW explained, “it forced us to get together and have a common goal for our project”.

### 3.3. Cognitive participation: proof of work

The SDH Summary—the clinic-specific summary of implementation decisions and plans—was used by some champions to offer proof to others (often clinic leadership) that work was indeed happening, and progress being made. As one champion and behavioral health director put it, it was a “tangible product” that “shows what we’ve been doing with these hours and not just … wasting time … that we’re really doing a meaningful thing”. In this way, the SDH Summary codified the cognitive participation that was happening in less visible forms.

In realist terms, the SDH Summary (M: resource) provided tangible evidence of progress and ongoing work that instilled confidence (M: reasoning) in clinic decision-makers that the process would result in meaningful change, thereby legitimatizing the work (O). Logic suggests that such an outcome would lead to continued support in the form of attention and dedicated time and/or resources (possible/future O). Champions that used the SDH Summary in this way tended to have leaders who were invested in increasing the number of social risk screenings and therefore paying attention to the work (C).

Clinics at which the champions shared the SDH Summary with leadership emphasized the importance of a tangible demonstration of progress—something that shows, as one champion and clinic manager put it, “we’re actually doing the work”. She further explained “all the work … was conversations at team meetings, one on one conversations with people, conversations at huddles … having something where I could … say look, this is what we’re doing … was really helpful!” Another champion and EHR specialist noted that “it was a great source of pride … to be able to … go hey look, no, no! This is what's happening. … And senior leadership is like oh awesome and they felt better that we were more forward than they thought we were. … And instead of it being a high priority and then dropped, no it's still a high priority, we are still working on it actively.”

### 3.4. Cognitive participation: relationships

The implementation support intervention set up three layers of relational interactions: between IST members, between the IST and clinic champions, and between the clinic champion(s) and clinic staff. Although the interactions were initially conceived primarily as a time for knowledge transfer, the tenor of these relationships had a profound impact on the clinic's engagement with the implementation support and continued drive toward change (NPT cognitive participation sub-construct: legitimation).

In realist terms, positive and respectful relationships that grew from the implementation support interactions (M: resource) fostered an environment in which clinic staff felt engaged and safe (M: reasoning#1) and therefore empowered and supported (M: reasoning#2) to be creative and experiment with workflows. This environment led to social risk screening workflows appropriately tailored to the specifics of the setting (O1) and therefore potentially more likely to be used and sustained (O2).

#### 3.4.1. Between IST members

While the two core IST members, the EHR trainer and practice facilitator, were initially chosen for their complementary expertise and competencies, their personal rapport established an enjoyable tone during clinic check-ins that set the stage for mutual learning. The two clearly appreciated each other as individuals and brought that warmth to their interactions with each other and the clinics (as one said, they bonded as “two old women in this young tech field”). Their rapport was explicitly noted by multiple clinic champions; as one remarked “Might seem small, but … You guys go so well together. We were joking, it's like these two knew each other their whole lives and they were best friends” and later “you enjoy your work … So that's the overall best part of it, it isn't the mechanics but how you two present it if that makes sense.” As the IST composition was not finalized until the beginning of the third (of six) intervention periods, the nine clinics randomized to the first two intervention periods did not experience the same backdrop to their implementation support.

#### 3.4.2. Between the IST and clinic champions

Clinic champions also called out the importance of the non-judgmental environment set by the IST, and the appreciation and respect showed for staff providing care in difficult circumstances, in creating a space in which they felt safe to ask questions and try and fail and try again. A champion and practice manager noted “one thing I really appreciated was the total non-judgmental bias … that you all stuck with me and my workflow and my process was SO appreciated … like you appreciated the work we were doing here too.”

Although the IST was unfailingly respectful, supportive and patient in all clinic interactions, there were variations in the tone of exchanges between clinics. In some instances the conversations felt warm, playful and interactive—one member of the IST observed about one clinic “I felt we built a relationship … day one everyone was best friends.” In others the tone was more business-like with a focus on pragmatic knowledge transfer and workflow guidance. Reasons for these differences varied—IST members had previous experience or relationships with certain clinics or individuals; some champions were constrained for time during the check-ins or clearly expected a tighter delivery of information; some seemed due to personality matches. In general, a sense of camaraderie between the IST and champions led to more brainstorming, co-construction and customization of screening workflows.

#### 3.4.3. Between clinic champion(s) and clinic staff

The relationship between clinic champion(s) and the rest of the clinic's providers and staff also influenced the extent to which staff felt engaged and supported in the work. What worked at a given clinic depended on its culture and context, and multiple approaches were successful in motivating and empowering staff to experiment with varied approaches and workflows. In some cases, champions were leaders with the authority to allocate time and space for collaborative work and to make workflow decisions; in others, champions were ostensibly lower in the clinic hierarchy (e.g., CHW, front desk staff) but came to the role with long-term trusting relationships in place at the clinic. One site was unwilling to name champions because, as they explained, they preferred to work collaboratively. This clinic was notable for the extent to which a group of staff, from program managers to medical assistants, solicited and listened to the perspectives and experience of different staff roles when designing their screening workflows; this also allowed them to pivot and increase social risk screening in the face of COVID-19.

Although staff roles and implementation approaches varied, champions who successfully fostered widespread engagement with the work were all highly respected and trusted by fellow staff *and* able to carve out time for themselves and others to devote to the work. Champions who had less social capital at the clinic, either because they were newer to their role or the organization, because they had not yet earned the trust of their colleagues, or because clinic culture afforded less respect to those in non-leadership positions, struggled to persuade others of the importance of collective action toward systematic social risk screening.

## 4. Discussion

Following the realist approach, we identified multiple pathways through which components of the implementation support intervention facilitated the establishment of workflows for the systematic collection of social risk information. Individual clinic outcomes were contingent on mechanism interactions and specific contextual factors that facilitated or inhibited this work. Three overarching program theories explained the mechanisms underlying why and how the intervention impacted the collection of social risk data. In brief: (1) Individual and collective views on the role of standardized social risk screening in “good” (relationship-based, patient-centered) care influenced receptivity to the intervention and uptake and sustainment of social risk screening. Those who saw value in standardization were motivated to engage with the support provided whereas those who saw standardization as a threat to human connection and rapport were likely to rebuff the implementation support. (2) The physicality of intervention materials (i.e., workbooks, summaries) anchored collaborative clinic discussions and acted as legitimizing “proof of work” for clinic champions. (3) Relationships between IST members, the IST and clinic champions, and clinic champions and staff were key drivers of uptake. Strong, respectful relationships empowered clinic staff to experiment with workflows that could work in their settings.

Despite robust program theories grounded in the qualitative data showing effective pathways toward change in specific contexts, the clinic-specific raw screening rates did not always demonstrate the expected uptake patterns. We argue that a lack of congruence between the study's and the clinics' definition of success accounts for this discrepancy, as described below.

First: While the trial's primary outcome measure was the rate of documented social risk screening, clinic staff cared most about meaningful engagement with and meeting the needs of individual patients. Screening rates are an imprecise indicator of these values, and structured social risk screening was seen as meaningful—and therefore worthwhile and warranting documentation—only if it was perceived to further person-centered care. We found, and prior research has demonstrated (42, 43), that EHR-based documentation of structured social risk domains can be perceived as a frustrating impediment to providing good care. As the program theories show, the support provided through the intervention did foster action in some clinics in some circumstances, but the value ascribed by the clinics to this work may have been less about screening *per se* and more about strengthening collective attention to contextualized, person-centered care. Simply put, some clinics viewed the support as successful in helping them improve contextualized care apart from formal screening. A story from one of the teams illustrates this point; though they clearly ascribed this outcome to their participation in the trial, it had little, if anything, to do with EHR-documented social risk screening.

*CHW: And we had a success story yesterday…*

*IST: … we'd love to hear.*

*CHW: So [provider] sent me a quick email about one of our patients … she said that he needed alcohol wipes, because he couldn't find any alcohol in the store for his diabetic stuff. So I called around to a couple different local resources and I was able to get alcohol wipes. But I couldn't get a hold of him so I came into the clinic and [MA] and I were able to talk. And I ended up just going to his house and dropping them off. We talked about all sorts of things and he was happy to put a face to my messages. And we're going to work together next week for SSI application and diabetic education with [provider]. I really enjoyed it because it was [provider], [MA] and I all working together to make sure the client successfully got what he needed.*

*IST: That is beautiful. Thank you for sharing that [CHW]. Congratulations. I bet that felt good.*

*[CHW]: It did feel good.*

*Program manager: It makes me want to cry! He's been a patient of ours for a long time and has so many challenges. It's just so nice to hear that little steps. You guys have really taken this and embraced it. You've taken an idea and process and really made it come to life.*

Second: Community-based health centers have sought to provide contextualized care since their inception; care informed by the particular challenges endemic to poverty is the core of what they do. Even when social risk screening is seen as a meaningful step toward contextualized person-centered care, and the implementation support is perceived as helpful in moving the clinic toward that goal, pragmatic constraints including staff churn, competing demands, limited time and lack of community resources to address patients' needs inhibit action. Third: Although many clinics began, as recommended in the intervention, with screening workflows addressing specific patient populations (e.g., new patients, patients with diabetes), for feasibility reasons the statistical analyses included all patients age 18 years and older. In some cases demonstrable progress may have been made in social risk screening rates for the specific patient groups, but these increases may have been obscured when lumped in with the larger patient pool.

NPT helped reconcile the realist program theories and trial outcome, and elucidated why the statistically significant increase in social risk screening rates during the intervention period was not sustained post-intervention. Although as demonstrated in the program theories many of the implementation support strategies were effective in many situations, the intervention as implemented fell more toward planning workflows than operationalizing and appraising them. In NPT terms, the implementation support addressed Coherence and Cognitive Participation, but did not move clinics to Collective Action and Reflexive Monitoring, and all four must be in place for a practice to become fully embedded (normalized, sustained) in a given setting. Thus it is possible that the strategies were appropriate but needed more time to take hold, i.e., the support intervention may have led to a sustained increase in social risk screening rates if the IST had more time to move clinics from planning to action. Six months is a relatively short period to expect change in a busy health center with multiple priority projects.

A second possibility is that the strategies chosen for the support intervention, especially those shown to be effective in the realist evaluation, emphasized preparation; alternate or additional support strategies might have been necessary to create long-term change. Or it may be the relationships built through the interactions fostered by the intervention, more than the individual strategies themselves, that made a difference (44). In this view the relationships between the clinics and the IST created excitement, momentum and accountability that led to an increase in screening rates, but when those supportive relationships ended new workflows were not yet fully integrated into care and were therefore not sustained in the face of pragmatic barriers to practice change (e.g., shifting priorities, staff turnover and burnout, lack of time).

Our understanding of these potential explanations is complicated by COVID-19, which forced a rapid, massive restructuring of care processes and priorities. Most of the participating clinics were on the front lines of testing and caring for patients with COVID-19; many quickly implemented telehealth and operated with a reduced workforce as providers and staff quarantined. While the forces at play in the realist program theories transcend COVID-19, it is undeniable that the pandemic exacerbated existing clinic challenges (e.g., staff churn) and rapidly transformed care priorities and delivery. Clinic staff consistently noted the impact of COVID-19 on their ability to implement systematic social risk screening—usually as a barrier to following through on planned screening workflow changes, but sometimes as the impetus for prioritizing this work in the face of so much obvious need.

In this study, realist evaluation and NPT brought complementary perspectives and were useful at different stages of the analysis process. We led with a realist-informed approach. Its ontological depth and related insistence on the importance of (and tools to help uncover) the underlying, often invisible mechanisms in explaining the movement (or not) toward change pushed us past surface explanations for our findings. Realist evaluation gave us a heuristic—the CMO configuration—to query, understand and explain variability and supported a focus on potential and possibility rather than on reductive measures of success / lack of success. NPT, on the other hand, emphasizes the empirical. As a middle range theory “strongly oriented to practice” (45) it asks: what is the work that people (collectively) need to do to enact new practice? Applying NPT to our existing program theories moved us toward a more broadly applicable explanation of why social risk screening rate increases did not sustain despite indications that the implementation support intervention was taking hold in some clinics—which increased the relevance and transferability to other interventions and settings. Leading with a realist-informed approach kept our insights sharp and grounded firmly in the data; applying NPT concepts in the initial phases of analysis would have felt overly prescriptive and restricted retroductive thinking. Layering in NPT was, however, critical in bringing the various threads into a cohesive, explanatory whole.

There are some important limitations to this analysis. Because our data sources were primarily observational we did not have consistent data on all topics of interest across clinics. The observational approach did ensure data were salient from the perspective of health clinics yet also meant that we did not always know how each clinic used or perceived each of the implementation support strategies (what triggered use of the workbooks as a collaborative tool, for instance, or how they did/did not share the SDH Summary with clinic leadership). We were also unable to request participant feedback on our emerging understandings, and thus missed an opportunity to refine and sharpen our program theories. The IST worked only with clinic staff, so the patient perspective is missing from the data and program theories. Participating clinics began the study with varied amounts of experience in social risk screening, from none to robust screening workflows, and this may have influenced receptivity to the implementation support provided. Finally, as noted above, it was difficult to appropriately account for the effects of COVID-19 on people's lives, values and work experiences.

Embedding new practices into existing routines requires shifts in mindsets as well as workflow. These findings emphasize the importance of framing, supporting and using social risk screening as a way to advance collective values of whole-person patient-centered care. Social risk screening is more likely to become normalized if clinic leadership clearly communicate their belief in its potential to build trust and rapport, enhance therapeutic alliances, improve patient-care team relationships and inform an individual's care, all of which result in better patient outcomes (46–61). To be effective, care teams must then be supported in putting these shared values into practice through dedicated time for collaboration and experimentation, for example, or staff training in difficult conversations. The details may vary—some clinics may choose to use the act of collecting the information to foster engagement, while others may choose to screen asynchronously (e.g., through patient portals or paper screens completed in the waiting room) and use the information to jumpstart deeper conversation—but placing relationship-building at the center of efforts to increase and sustain social risk screening may be key to long-lasting success. Similarly, these results highlight the importance of implementation strategies that draw on and support trusting relationships as mechanisms of change. This may be particularly important in social determinants of health work, which requires commitment and humility from health care providers and vulnerability on the part of patients.

## Data Availability

The datasets presented in this article are not readily available because of regulatory requirements for participant privacy and confidentiality. Requests to access the datasets should be directed to buncea@ochin.org.
