# Supplementary material for: Finding meaning: a realist-informed perspective on social risk screening and relationships as mechanisms of change
Source: Front Health Serv. 2023 Oct 23;3:1282292. doi: 10.3389/frhs.2023.1282292 (PMC10626542; doi:10.3389/frhs.2023.1282292)
Supplement: Supplementary file 1 [file Table1.docx]

ASCEND Clinic Summary Template

**Clinic, Organization**

**Intervention period [date]**

**Setting**

[*# of participating clinic/# of total clinics in organization; location*]

**Context**

General, big picture context

[*bullet points*]

Big picture context specific to time period of implementation

[*bullet points*]

Project-specific context

[*bullet points*]

*Include:*

Champion(s): name, staff role, involvement in SDH workflow

Others who regularly attended calls: name, staff role, involvement in SDH workflow

SDH workflow / work prior to ASCEND implementation

SDH workflow at end of wedge, planned or in process

**Intervention characteristics**

[*what assistance requested (apart from usual implementation), and what fulfilled – e.g., support emails; adaptations and customizations to intervention that provided to clinic*]

Relationship between support team and clinic staff

*[as relevant; note if based on data or information gained elsewhere]*

**Mechanisms**

[focus on how and why]

[*include interpretations, noted as such; note gaps in our data, including a preponderance of either reasoning or behavioral data*]

**Resources**

[*materials created by clinic, e.g., clinic screener translations*]
