# Supplementary material for: Finding meaning: a realist-informed perspective on social risk screening and relationships as mechanisms of change
Source: Front Health Serv. 2023 Oct 23;3:1282292. doi: 10.3389/frhs.2023.1282292 (PMC10626542; doi:10.3389/frhs.2023.1282292)
Supplement: Supplementary file 2 [file Table2.docx]

**ASCEND post-wedge member checking short survey**

[Insert clinic specific summary of our learnings, barriers, and facilitators]

The above is a summary of the primary factors influencing the implementation of systematic SDH screening at your clinic based on data collected during the ASCEND check-in calls.

1. Are there additional key factors that influenced the implementation of SDH screening at your clinic that are not captured in our summary? Please explain.
2. In the 6 months after the ASCEND implementation support ended [dates], were there any changes that impacted your clinic’s ability to further implement and conduct systematic SDH screenings? Please explain.
3. In the 6 months after the ASCEND implementation support ended [dates], were there defining moments in which your clinic’s ability to systematically screen for SDH changed for the better or the worse? Please explain.
4. Optional: Please share anything else you think might help us understand the barriers and facilitators to implementing systematic SDH screening at your clinic.
